# Development of an Assessment for Entrustable Professional Activity (EPA) 10: Emergent Patient Management

**DOI:** 10.5811/westjem.2016.10.31479

**Published:** 2016-12-05

**Authors:** Laura R. Thompson, Cynthia G. Leung, Brad Green, Jonathan Lipps, Troy Schaffernocker, Cynthia Ledford, John Davis, David P. Way, Nicholas E. Kman

**Affiliations:** * The Ohio State University College of Medicine, Department of Emergency Medicine, Columbus, Ohio; † The Ohio State University College of Medicine, Department of Internal Medicine, Columbus, Ohio; ‡ The Ohio State University College of Medicine, Department of Emergency Anesthesiology, Columbus, Ohio

## Abstract

**Introduction:**

Medical schools in the United States are encouraged to prepare and certify the entrustment of medical students to perform 13 core entrustable professional activities (EPAs) prior to graduation. Entrustment is defined as the informed belief that the learner is qualified to autonomously perform specific patient-care activities. Core EPA-10 is the entrustment of a graduate to care for the emergent patient. The purpose of this project was to design a realistic performance assessment method for evaluating fourth-year medical students on EPA-10.

**Methods:**

First, we wrote five emergent patient case-scenarios that a medical trainee would likely confront in an acute care setting. Furthermore, we developed high-fidelity simulations to realistically portray these patient case scenarios. Finally, we designed a performance assessment instrument to evaluate the medical student’s performance on executing critical actions related to EPA-10 competencies. Critical actions included the following: triage skills, mustering the medical team, identifying causes of patient decompensation, and initiating care. Up to four students were involved with each case scenario; however, only the team leader was evaluated using the assessment instruments developed for each case.

**Results:**

A total of 114 students participated in the EPA-10 assessment during their final year of medical school. Most students demonstrated competence in recognizing unstable vital signs (97%), engaging the team (93%), and making appropriate dispositions (92%). Almost 87% of the students were rated as having reached entrustment to manage the care of an emergent patient (99 of 114). Inter-rater reliability varied by case scenario, ranging from moderate to near-perfect agreement. Three of five case-scenario assessment instruments contained items that were internally consistent at measuring student performance. Additionally, the individual item scores for these case scenarios were highly correlated with the global entrustment decision.

**Conclusion:**

High-fidelity simulation showed good potential for effective assessment of medical student entrustment of caring for the emergent patient. Preliminary evidence from this pilot project suggests content validity of most cases and associated checklist items. The assessments also demonstrated moderately strong faculty inter-rater reliability.

## INTRODUCTION

In 2014, the Association of American Medical Colleges (AAMC) published 13 Core Entrustable Professional Activities, or EPAs, considered essential competencies medical school graduates are expected to be able to perform prior to entry into residency.^1^ EPAs are considered foundational for all practicing physicians, regardless of specialty choice and describe sets of integrated competencies required for the care of specific patient types in specific patient settings. EPAs are multifaceted and integrated, making them more appropriate to assess holistically.^2^

As originally conceived, entrustment was a discrete standard that literally meant that a graduated medical student was prepared to perform a patient care activity without direct supervision. This has been debated as too ambitious for some patient types and some care settings. Chen et. al. have proposed an alternative framework of entrustment for undergraduate medical education (UME) to include a continuum of UME entrustment and supervision.^3^

If conceived as a discrete standard, EPA-10 poses substantial challenges for undergraduate medical educators, both logistically and ethically. This is the type of EPA Chen addresses when suggesting that entrustment should be considered a continuum rather than discrete. EPA-10 requires medical students to “recognize a patient requiring urgent or emergent care and initiate evaluation and management.”^1^ To earn entrustment, a student must recognize a patient’s clinical decompensation or abnormal vital signs, gather information to determine possible causes, begin initial stabilization, and call for assistance. Challenges involve the lack of opportunities students have to manage patients requiring emergent care. Even when a student does encounter an emergent patient, concern for patient safety often precludes their involvement in the patient’s evaluation and management. Consequently, alternative methods for assessing EPA-10 and perhaps the adoption of a continuum of entrustment and supervision are needed.^3^, ^4^

The purpose of this project was to develop an assessment method and associated instrumentation for evaluating medical students on EPA-10. The project involved the design of case-scenarios representing patients in need of emergent care, the design of high-fidelity simulations to evaluate the student’s performance on these cases, and the design of an assessment instrument for faculty to document the student’s performance.

## METHODS

### Educational Program (Setting)

Our population was fourth-year medical students (M-4s) at The Ohio State University College of Medicine. Our class sizes average about 190 students per year. M4s at our institution must complete several required rotations, one of which is a one-month emergency medicine (EM) clerkship. We defined our study period as June-December 2015, which provided sufficient numbers of EM clerkship students to evaluate the EPA-10 assessment method. By selecting this time period, we were also assured that we captured performance data for medical students who were most likely going into EM. The goals of the EM clerkship are to attain knowledge about the practice of emergency medicine and to build skills in the assessment and management of the undifferentiated patient. The EM clerkship enrolls an average of 20 students per month. During the clerkship students work 120 clinical hours at one of seven regional emergency departments. They participate in three hours of didactics; workshops on airway, suturing, IV placement, and ultrasound; and complete 10 online learning modules. Students prepare for the EPA-10 assessment through clinical work and the completion of study modules from the National (U.S.) EM Clerkship Curriculum.^5^

### Case Development

A team of experts in both emergent care and medical student education wrote scripts for five case scenarios involving an unstable patient requiring resuscitation. Cases were derived using the criteria set forth in the AAMC’s Core EPAs for Entering Residency: Curriculum Developers Guide.^1^ The five case scenarios were developed on the basis of their general prevalence, unstable presentations, and easily observed critical actions required for establishing a medical and/or surgical plan. Each scenario was designed to address two or more of the medical conditions recommended by the AAMC Core EPA document.^1^ The cases were written using classical illness scripts so diagnosis should have been relatively clear to a fourth-year medical student, leading to a diagnostic and therapeutic plan with which they are familiar. The cases are listed here and in Table 1:

1. Chest pain: unstable atrial fibrillation (Afib)
2. Abdominal pain: ruptured ectopic pregnancy (REP)
3. Confusion and fever: sepsis (SEP)
4. Headache: subarachnoid hemorrhage (SAH)
5. Trauma and shortness of breath: trauma-related tension pneumothorax (PTX)

### Simulation Development

High-fidelity simulation was chosen as the modality for the EPA-10 assessment because it provided a balance between the realistic portrayal of an unstable patient and standardization across cases and assessment sessions. Cases were forwarded to local simulation experts: an emergency physician who was fellowship trained in simulation and three simulation technicians from our Clinical Skills Education and Assessment Center (CSEAC). These individuals crafted a simulation for each case. Simulations were designed to take place in replicas of ED resuscitation bays in our CSEAC. Each bay was equipped with a programmable simulation manikin, and staffed by a faculty facilitator/evaluator, a simulation technician, and a nurse confederate. Voices of the patient, family and other healthcare team members were provided through telephone or speakers.

### Evaluation Instrument Development

An expert panel of education faculty (three EM, one Anesthesia) was tasked with developing the assessment instrument for evaluating student performance on each simulated case. The AAMC EPA Curriculum Developers Guide was again used to identify expected performance tasks for a learner who would be entrusted to recognize a patient requiring emergent care; initiate evaluation and management; and seek help within the clinical contexts assessed.^1^

The performance tasks were designed to be highly observable and low inference. Accordingly, they were converted to three types of checklist items. First, a set of three universal critical actions were identified and applied to all cases: *recognizes unstable vital signs*; *asks for help*; and *determines patient’s disposition*. These were supplemented with case-specific critical actions related to 1) identification of underlying etiologies of the patient’s decompensation, 2) initiation of care plans, and 3) application of basic and advanced life support. Finally, each case included a global entrustment item that asked whether the evaluator would “entrust” the student to manage a similar case unsupervised.

Checklist items were reviewed and revised by experts in critical care, simulation design and assessment (Table 2). The instruments were formatted for use in a web-based electronic assessment platform called Myprogress^TM^ and were delivered for use by faculty through wireless tablet computers. Performance data were collected and stored until needed in the Myprogress^TM^ cloud-based computer servers.^7^

### Assessment Method

Prior to the simulation, students were provided an orientation to the trauma bay. For each case, they were provided a chief complaint and instructed to treat the manikin as a real patient. Simulations were designed for teams of four students. Each team completed four 30-minute cases. Each student was designated as team leader for one case. As team leader, they were responsible for making all medical decisions, recognizing critical actions and assigning tasks to the other team members. Student performance was evaluated only during their turn as team leader. Faculty facilitators completed the EPA-10 evaluation checklists in real time during the simulation.

All decisions made during the case, including medications administered or procedures performed, altered the course of the case based on pre-programmed simulator responses to each action. If a team leader failed to perform a critical action during the simulation, the nurse confederate provided prompts to move the case along. For example, if the student failed to initiate IV fluids in a hypotensive patient, the nurse might say “I am worried about this patient. His blood pressure seems very low.” The nurse would give additional prompts as needed until the critical action was performed. When the team leader gave an unanticipated order, the controllers improvised or altered the simulation to follow that directive. A log of improvised alterations was kept so that consistent responses could be programmed into the simulation for future assessments.

During the study period, the EPA-10 assessment was considered a formative evaluation. Students were only required to participate and receive formative feedback on their performance. Students who performed poorly (did not attain the rating of “global entrustment”), were offered a coaching session during which they were provided a chance to perform additional cases.

To evaluate inter-rater reliability for the assessments, we scheduled two faculty facilitator/evaluators for each trauma bay during the first two months of the project. After that, scheduling two faculty per trauma bay became cost prohibitive.

### Scoring

Performance data was downloaded from Myprogress^TM^ and scored. We scored each critical action item as “YES”, “NO” or “With prompting from the nurse confederate.” For analysis purposes, the “With prompting” rating was rescored as a “NO” since the performance did not meet the threshold of being executed autonomously. Global entrustment was assessed as “YES” or “NO.”

### Analysis

Besides descriptive statistics, we conducted three primary analyses to investigate the psychometric properties of the EPA-10 instruments. We used the Krippendorf ‘s alpha (K-Alpha) statistic to evaluate inter-rater reliability among the faculty evaluators.^8^,^9^ The K-Alpha provides stable estimates of inter-rater reliability under the conditions of partially-crossed designs. (Partially-crossed designs occur when all subjects are not evaluated by all judges.)^8^ It has become the most recommended measure of inter-rater reliability with nominal level data like yes-no checklists.^10^–^13^ We calculated K-Alphas for each checklist item, including the global entrustment rating.

We calculated tetrachoric correlations (R_tet_) between each checklist item and its corresponding global entrustment item. The R_tet_ provides an indicator of internal consistency within the checklist. A high R_tet_ also implies that the item contributes to the global entrustment decision.

To evaluate inter-rater reliability of faculty pairs on their global entrustment ratings, we calculated the percent agreement and Cohen’s kappa coefficients across all subjects. In situations involving dichotomous data and pairs of raters, Uebersax recommends using the p-values from calculating Cohen’s kappa coefficients to assess whether agreement exceeds that which might be expected by chance.^10^,^13^ The results of this test informed us about which pair of raters had the best agreement and which require additional feedback or training.

We did most computations using SPSS for Windows, V. 22.^14^ The Krippendorff’s alpha measures were calculated using an SPSS syntax module written by Hayes.^15^ We calculated the tetrachoric correlations using an SPSS syntax module called Tetra-Com.^16^ This project was determined to be exempt from humans subjects review by our institutional review board.

## RESULTS

One hundred fourteen medical students, or 62% of the total class (114 of 185) participated in the EPA-10 assessment between June and December of 2015. Table 3 summarizes the number of students by rotation, case scenario, and number of evaluators. Three cases were used for every rotation: Afib (30, 26%), SEP (26, 23%), and PTX (28, 25%). A fourth case (SAH) was determined to be too easy and was subsequently replaced with the ruptured ectopic pregnancy case (REP). Due to a technical problem with the web-based assessment platform used for data collection, the data for the REP case were incomplete. Twenty-eight percent of students were evaluated by more than one faculty member (32 of 114).

Almost 87% of the students were rated as having reached ad-hoc entrustment as defined by the EPA-10 criteria (86.8%; 99 of 114). Cohen’s kappa coefficients across the four pairs of judges who jointly assessed students on global entrustment ranged from 0.46–1.0, with three of the four pair’s agreement being significantly better than chance. Two of the Kappa coefficients show substantial agreement, while the other two show moderate agreement (Table 4).

The K-Alpha inter-rater reliabilities allowed us to look at faculty agreement on global entrustment for each case. The K-Alpha values were 0.53 for the Afib case, 0.61 for the PTX case and 1.00 for the SEP case. We were unable to calculate a K-Alpha value for the SAH case since all evaluators selected the same response; however, this implies perfect inter-rater reliability.

### Summary of Common Critical Action Items

Three critical action items were common to all four cases: *Obtains & recognizes patient status–unstable vital signs, Asks for help when needed* and *Determines patient disposition*. All students were rated as entrusted by all raters for the SAH case. Consequently, we were unable to calculate the R_tet_ coefficients for these items.

All but two students achieved entrustment on the first item “*Obtains & recognizes patient status–unstable vital signs*,” across all cases. For the students who were evaluated by two faculty, inter-rater agreement was near perfect. Accordingly, there was little information gleaned from the statistical analyses for this critical action item. We did observe, however, high positive and significant R_tet_ correlations with the global entrustment outcome for both SEP and PTX cases.

The *Asks for help* item suffered from poor inter-rater agreement on the Afib and SEP cases. Raters demonstrated better agreement on the other two cases, SAH and PTX. R_tet_ correlations with the outcome can be considered strong for the Afib and PTX cases, and low but positive for the SEP case.

Faculty raters generally agreed on whether students “*Determined patient disposition*” for three of the four cases. The exception was the PTX case, which suffered a negative K-Alpha value (−.083). R_tet_ correlations for this item were positive across three cases: 0.29 for Afib, 0.49 for PTX and 0.63 for the SEP case.

### Summary of Stabilizing Treatment Items

The case instruments contained between three and five case-specific “*stabilizing treatment items.*” With a few exceptions, these items generally performed well, meaning there was positive and substantial inter-rater agreement and strong, positive R_tet_ correlations with the EPA-10 outcome rating of each case.

Poor inter-rater agreement was observed on two of the items within the SAH case: lumbar puncture (−.083) and calling for a neurosurgery consult (−.167). For the SEP case, poor inter-rater agreement was observed for installation of a central line (−.222). Finally, there was also lack of inter-rater agreement on the PTX case for establishing an airway and rechecking vital signs (−.083).

### Summary of Cases

Missing data posed a minor problem for this study. A complete evaluation of the REP case was not possible due to a technical problem. Evaluator ratings of the items on the SAH case lacked variability so that statistics were impossible to calculate, leaving it difficult to interpret item performance. The other three cases suffered some missing data, but were still able to be evaluated. For the Afib and PTX cases, all items were observed to have positive R_tet_ correlations with global entrustment. The SEP case, however, consisted of two items that did not have strong correlations with global entrustment. One was due to lack of variability in the ratings. (Every subject was scored as having achieved that critical action.) The other had a positive, but low R_tet_ correlation (.19) with global entrustment.

## DISCUSSION

Entrustable professional activities represent an important addition to the framework of modern medical training. Measurement of these essential activities contributes to certifying a trainee’s ability to perform to accepted standards of care. Medical schools and residency programs have a responsibility to the public to assure that their graduates have been assessed for entrustment of these activities prior to unsupervised practice. To meet this responsibility, medical educators must integrate high-quality, formal EPA assessments into their training programs.

EPA-10 is particularly important because it requires the medical student to recognize an unstable patient who requires life-saving, emergent care. Assessing a medical student’s ability to perform EPA-10 activities is difficult in the clinical setting. High-fidelity simulation (HFS) offers the opportunity to train and assess medical students on EPA-10 related competencies. Literature on the use of HFS for assessing EPA-10 is limited; however, residents at some Canadian institutions have been effectively assessed with checklists and HFS.^17^^,18^

Three critical actions were common across all of the cases: *recognizing abnormal vital signs*, *asking for help when needed*, and *determining patient disposition*. Reassuringly, only two students failed to recognize abnormal vital signs. Disconcerting, however, is that 20% of students (23 of 114) failed to ask for help when needed, and 22% (25 of 114) failed to accurately determine the patient disposition. The first common item achieved strong inter-rater reliability, probably because of well-established parameters and clearly defined values for vital signs. The other two common items had inconsistent inter-rater reliability across cases. They had good agreement on *determining disposition plan* in three of the four scenarios, excluding PTX. We believe that the low inter-rater reliability on the disposition item of the PTX case was primarily due to variable approaches to airway management across evaluators from two different specialties. For *asking for help,* the inconsistency in inter-rater reliability two of four cases (Afib, and SEP). This was likely due to inconsistency in how faculty interpreted the student behaviors.

For the checklist rating scale instruments, we attempted to maximize inter-rater reliability by selecting performance tasks that were 1) highly observable (a rater would know “it” when they see “it”) and 2) low inference (easily interpreted). The prompting from the nurse confederate was needed to complete the simulation in the allotted time. However, for measurement purposes, the rating of “with prompting” became a source of unreliability. For all statistical tests, we recoded this value to a “NO” response, indicating that the student had not reached a measurement threshold of entrustment. We believe that the use of this rating scale option was a source of inconsistency among our raters (i.e., some raters used this rating frequently, and others used it not at all). In the future, this rating will have to be more clearly defined or eliminated from the instrument. A good example of inter-rater reliability measures affected by this problem occurred in the SAH case, items 6 and 7 and SEP case, item 6.

Overall, we found that nearly 87% of students met our global assessment of *ad hoc* entrustment. Additionally, we observed good inter-rater reliability among the four pairs of established faculty raters on this global entrustment item. We did not specifically measure the impact of team support on the team leader’s entrustment; however, this most certainly affected determination of global entrustment for some students.

For the Afib and PTX cases, all items were observed to have high, positive R_tet_ correlations with global entrustment. We interpret this to mean that these items contribute significantly to the entrustment decision and are important components of the measurement instrument. The SEP case, however, consisted of two items that did not have strong correlations with global entrustment, one due to lack of variability in this outcome. (Every subject was scored as having achieved that critical action.) The other had a positive but low correlation with global entrustment. We believe that these two items need to be revised or replaced to improve their ability to discriminate between high- and low-performing students.

## LIMITATIONS

We confronted several limitations. First, a complete evaluation of the REP case was not possible due to significant missing data points caused by a technical glitch in the electronic data collection platform. This case will have to be re-evaluated in the future. Second, we were unable to completely isolate an individual student’s performance from the performance of the team. Conversely, there was no way to recognize an underperforming team leader who performed well in their support role during another scenario. A third limitation is derived from the logistics of our assessment methods. Since students participated in more than one case but were only evaluated on the case they led, there could have been a cumulative practice benefit for the students who were last to lead. In the future we would like to measure the practice effect obtained by repeated participation in simulated case scenarios such as those used for this project.

Limitations on generalizability to other medical schools may include equipment availability, time investment of faculty and support staff. HFS equipment and qualified technical support staff require a significant institutional monetary investment. For each student assessment we used 1–2 trained physician faculty raters, a trained simulator specialist, and a trained actor for the resuscitation bay nurse role. Each assessment lasted up to 30 minutes per student. Substantial cost-savings might be realized by the use of trained non-physician evaluators.

Future research is needed to establish how well *ad hoc* entrustment based on a single simulation case can predict entrustment in the care of actual patients.

## CONCLUSION

We have designed an evaluation for EPA-10 that includes universal critical actions, case-specific critical actions, and a global rating of ad-hoc entrustment. The preliminary evidence suggests that inter-rater reliability and content validity were achieved for three of four case simulations and checklist instruments. Future studies are needed to establish generalizability across other patient cases and other institutions.

## Figures and Tables

**Table 1:** Summary of five case scenarios used for assessment of entrustable professional activity (EPA 10) in medical students.

Case	Patient conditions	Critical actions
1. Chest pain: Atrial fibrillation (Afib)	Arrhythmia, chest pain, hypotension	Obtain a 12-Lead EKG Initiate medical management (Beta-blocker or CCB) Cardiovert the unstable patient
2. Abdominal pain: ruptured ectopic pregnancy (REP)	Hypotension, tachycardia, mental status change	Start IV fluid bolus Transfuse O neg. blood Perform pelvic ultrasound or FAST exam Consult OB/Gyn
3. Confusion and fever: sepsis (SEP)	Hypotension, fever, mental status change	Order IV fluid bolus Order antibiotics Establish central line access Start pressors
4. Headache: subarachnoid hemorrhage (SAH)	Mental status change, hypertension	Order head CT Perform lumbar puncture Consult neurosurgery Administer IV anti-hypertensive medication
5. Trauma and shortness of breath: trauma related tension pneumothorax (PTX)	Chest pain, shortness of breath, hypotension, tachycardia	Perform primary survey (ABCs) Perform needle thoracostomy Order CXR Reassess the patient

*EKG,* electrocardiogram; *CCB,* calcium channel blocker; *IV,* intravenous; *CT,* computed tomography; *CXR*, chest x-ray

**Table 2:** Inter-rater reliability, Tetrachoric correlations (R_tet_), and frequencies and (percentages) of judge ratings.

			Judge 1		Judge 2	
				
	K-alpha	R_tet_ (n=41)	No	Yes	No	Yes
Case 1: Chest pain: atrial fibrillation (Afib) (30 subjects, 4 judges with 8 overlapping)						
1.Obtains and recognizes patient status - unstable vital signs	NA	-	0	30 (100)	0	8 (100)
2. Asks for help when needed	−.083	.75†	3 (10)	27 (90)	0	8 (100)
3. Determines patient disposition*	1.00	.29†	3 (10)	26 (87)	1 (13)	7 (88)
4. Provides stabilizing treatment: obtain 12 lead EKG	1.00	.40†	5 (17)	25 (83)	3 (38)	5 (63)
5. Provides stabilizing treatment: beta blocker or CCB	.762	.51†	15 (50)	15 (50)	6 (75)	2 (25)
6. Provides stabilizing treatment: when vitals change cardioversion w/o consent	.458	.81†	7 (23)	23 (77)	4 (50)	4 (50)
Global EPA-Afib: meets entrustment	.531		6 (20)	24(80)	5 (63)	3 (38)
Case 3: Confusion and fever: sepsis (SEP) (26 subjects, 4 judges with 8 overlapping)						
1. Obtains and recognizes patient status - unstable vital signs	0.00	.80†	0	26 (100)	1 (14)	6 (86)
2. Asks for help when needed*	−.182	.19†	2 (8)	22 (92)	2 (29)	5 (71)
3. Determines patient disposition*	.571	.63†	1 (4)	22 (96)	2 (29)	4 (57)
4. Provides stabilizing treatment: IVF bolus	NA	.00†	0	26(100)	0	7 (100)
5. Provides stabilizing treatment: ABTCS	1.00	.87†	1 (4)	25 (96)	1 (14)	6 (86)
6. Provides stabilizing treatment: central line	−.222	.56†	9 (35)	17 (65)	5 (71)	2 (28)
7. Provides stabilizing treatment: pressor	.313	.51†	3 (12)	23 (89)	3 (43)	4 (57)
Global EPA-sepsis: meets entrustment	1.00		3(12)	23 (89)	2 (29)	5 (71)
Case 4: Headache: subarachnoid hemorrhage (SAH) (11 subjects, 4 judges with 7 overlapping)						
1. Obtains and recognizes patient status - unstable vital signs	0.00	-	1 (9)	10 (91)	1 (14)	6 (86)
2. Asks for help when needed	1.00	-	2 (18)	9 (82)	1 (14)	6 (86)
3. Determines patient disposition	.606	-	3 (27)	8 (73)	1 (14)	6 (86)
4. Provides stabilizing treatment: pain control	1.00	-	5 (46)	6 (55)		5 (71)
5. Provides stabilizing treatment: CT head*	NA	-	0	11 (100)	0	7 (100)
6. Provides stabilizing treatment: lumbar puncture	−.083	-	0	11 (100)	2 (29)	5 (71)
7. Provides stabilizing treatment: consult neurosurgeon*	−.167	-	1 (9)	8 (73)	1 (14)	3 (43)
8. Provides stabilizing treatment: admin IV antihypertensive*	NA	-	0	6 (55)	0	1(14)
Global EPA-SAH: meets entrustment	NA	-	0	11(100)	0	7 (100)
Case 5: Trauma and shortness of breath: trauma/tension pneumothorax (PTX) (28 subjects, 4 judges with 8 overlapping)						
1. Obtains and recognizes patient status - unstable vital signs*	NA	.68†	0	28 (100)	0	8 (100)
2. Asks for help when needed	.350	.71†	10 (38)	18 (64)	3 (38)	5 (63)
3. Determines patient disposition*	−.083	.49†	9 (32)	19 (68)	5 (63)	3 (38)
4. Provides stabilizing treatment: airway and vitals	−.083	.87†	2 (7)	26 (93)	1 (13)	7 (88)
5. Provides stabilizing treatment: needle thoracostomy	1.00	.70†	8 (29)	20 (71)	2 (25)	6 (75)
6. Provides stabilizing treatment: x-ray and reassess	1.00	.52†	2 (7)	26 (93)	2 (25)	6 (75)
Global EPA-PTX: meets entrustment	.606		5 (18)	23 (82)	1 (13)	7 (88)

* Values were missing from this variable due to software problems.

† Statistically significant.

NA = Judges have perfect agreement using the same rating for all subjects. i.e. The Krippendorf’s alpha value is indeterminate when all judges rate all subjects with the same score.

*EKG,* electrocardiogram; *CCB,* calcium channel blocker; *EPA,* entrustable professional activity; *IVF,* intravenous fluid; *CT,* computed tomography; *IV,* intravenous

**Table 3:** Number of medical student participants and faculty evaluators by rotation, along with number and percentage of those who attained entrustment (i.e., met EPA).

		Number of students evaluated with each case						Students evaluated by how many faculty		
			
Rotation	Afib	REP	SEP	SAH	PTX	TOTAL	Met EPA	One	Two	Three
1	3	0	3	3	3	12	9 (75%)	0	0	12
2	4	0	3	4	4	15	11 (73%)	0	15	0
3	5	0	4	4	4	17	17 (100%)	17	0	0
4	5	6	5	0	6	22	20 (90%)	22	0	0
5	6	6	6	0	5	23	21 (92%)	18	3	0
6	4	4	3	0	3	14	10 (71%)	14	0	0
7	3	3	2	0	3	11	11 (100%)	11	0	0
TOTAL	30	19	26	11	28	114	99 (87%)	82	18	12

*Afib,* atrial fibrillation; *REP,* replaced*; SEP,* sepsis; *SAH,* subarachnoid hemorrhage, *PTX,* pneumothorax; *EPA,* entrustable professional activity.

**Table 4:** Pairwise percentage agreement (upper diagonal) and Cohen’s kappa coefficients (lower diagonal) for judgments on 31 subjects on entrustment, or the student’s ability to manage an acutely decompensating/acutely ill patient with a life-threatening illness. (Note: The number of students rated jointly by the judge pair are in parentheses in the upper diagonal.)

Faculty raters	A	B	C	D	Summary
A		100(8)	-	-	100(8)
B	1.00**		81(16)	85(13)	83(29)
C	-	.46		90 (20)	90(20)
D	-	.57*	.73***		

* Significant at p<.05

** Significant at p<.01

*** Significant at p<0.001

Key: Cohen’s kappa coefficients: <0.0 = poor, less than chance agreement; 0.01 to 0.20 = slight agreement; 0.21 to 0.40 = fair agreement; 0.41 to 0.60 = moderate agreement; 0.61 to 0.80 = substantial agreement; 0.81 to 0.99 = almost perfect agreement.
